# Molecular epidemiology of a multidrug-resistant Shigella sonnei outbreak in Tunisia (2022–2023) using whole-genome sequencing

**DOI:** 10.1099/mgen.0.001362

**Published:** 2025-03-06

**Authors:** Fahmi Smaoui, Boutheina Ksibi, Senda Mezghani, Eya Guermazi, Fatma Charfi, Sonia Ktari, Nourelhouda Ben Ayed, Thouraya Kammoun, Héla Karray, Adnene Hammami

**Affiliations:** 1 Research Laboratory Microorganisms and Human Disease 'MPH LR03SP03', Laboratory of Microbiology, Habib Bourguiba University Hospital, Sfax, Tunisia; 2 Laboratory of Microbiology, Faculty of Medicine of Sfax, University of Sfax, Sfax, Tunisia; 3 Pediatric Department, Hedi Chaker University Hospital, Sfax, Tunisia

**Keywords:** multidrug resistance, *Shigella sonnei*, Tunisia, whole-genome sequencing

## Abstract

**Purpose.** The prevalence of multidrug-resistant (MDR) *Shigella sonnei* is increasing globally, raising concerns for public health. In 2022, an outbreak of MDR *S. sonnei* was observed in Tunisia. We aimed to evaluate the genetic profile of *S. sonnei* isolates during the outbreak, including their clonal relationship, antimicrobial determinants and connection to international strains.

**Methods.** In this study, we sequenced the whole genome of 24 S. *sonnei* strains collected from South Tunisia between July 2022 and November 2023. Bioinformatic analysis was conducted to confirm species identification, assign sequence types, determine core genome sequence types, analyse phylogenetic relationships and identify antimicrobial resistance determinants. Phylodynamic and phylogeographic analyses were performed to trace the spatiotemporal spread of the outbreak genotype.

**Results.** Our investigation revealed that 23 out of 24 isolates were grouped into the HC10-20662 genotype within the 3.6.3 subclade. All isolates carried the *bla_CTX-M-15_* gene associated with extended-spectrum beta-lactamase production, as well as the *dfrA1* and *qnrS1* genes, along with the D87G mutation in *gyrA*. Additionally, the s*ul2, tet(A*) and *mph(A*) resistance genes were present in most isolates (96%, 96 and 83, respectively). Phylogeographic analysis suggested that the outbreak genotype likely spread in Europe before being introduced into Tunisia.

**Conclusion.** To the best of our knowledge, this is the first MDR *S. sonnei* outbreak in the country. The HC10-20662 genotype appears to be responsible for a multi-country outbreak, affecting both Tunisia and Europe. Continued genomic surveillance efforts, both nationally and internationally, are essential for monitoring the dynamic evolution and global spread of MDR * S. sonnei*.

**Impact Statement**

Multidrug-resistant (MDR) *Shigella* represents a significant and emerging global public health threat and is designated as a priority bacterial pathogen by the World Health Organization. During 2022–2023, Tunisia reported its first outbreak of MDR shigellosis, resulting in numerous severe cases, including one confirmed fatality. This outbreak was caused by *Shigella sonnei*, a species not historically prevalent in the country. Our study employed whole-genome sequencing to characterize *Shigella* strains from South Tunisia, identifying the responsible genotype as HC10-20662 within the 3.6.3 subclade. Most strains within this genotype exhibited similar phenotypic and genomic resistance profiles. Our study tracked the spatial and spatiotemporal dynamics of this genotype, revealing its spread in Europe before its introduction to Tunisia. This research marks the first genomic analysis of *Shigella* in Tunisia and should provide valuable insights for public health decision-makers in both Tunisia and Europe. Continuous genomic surveillance of this genotype and other emerging MDR *Shigella* clones remains essential for preparedness and timely response against potential future shigellosis outbreaks.

## Data Summary

The sequencing data generated from this study were deposited under BioProject accession number PRJNA1111155 in the NCBI BioProject database. Their Sequence Read Archive accession numbers and metadata are available in Table S1, available in the online Supplementary Material. The metadata of international sequences that were retrieved from EnteroBase are available in Table S2. Tables S1 and S2 are provided as a supplementary Excel file in the online version of this article. Additionally, four figures (Figs S1, S2, S3 and S4) are provided as supplementary materials. All supporting data, code and protocols have been provided within the article or in supplementary data files.

## Introduction

*Shigella* is a significant cause of bacterial gastroenteritis worldwide, being responsible for ~80–165 million cases worldwide [1]. Shigellosis is the second leading cause of diarrheal deaths globally, resulting in ~160 000 fatalities annually, with the majority occurring in young children in low- and middle-income countries [2,3]. This highly contagious disease spreads through faecal-oral contact, with an exceptionally low infectious dose, ranging from 10 to 100 cells [4]. Humans serve as the natural reservoir for *Shigella* species [5].

*Shigella* is a Gram-negative *Enterobacterales* that comprises four distinct species: *S. boydii, S. dysenteriae, S. flexneri* and *S. sonnei* [6]. Historically, *S. sonnei* is the most frequent agent of the disease in high-income countries and is subject to public health surveillance [7]. In recent years, there has been an increasing number of reports of multidrug-resistant (MDR) *S. sonnei* outbreaks in many regions worldwide [8,12]. The rise in MDR among *Shigella* spp. has elevated its status as a World Health Organization priority pathogen, emphasizing the urgent need for effective surveillance and control measures [13]. Understanding the dynamics of MDR *S. sonnei* outbreaks is crucial for implementing effective control and prevention measures to mitigate their impact on public health, particularly in the context of increasing antimicrobial resistance.

Whole-genome sequencing (WGS) offers unprecedented resolution in characterizing bacterial isolates, enabling an in-depth investigation of the genetic diversity, transmission patterns and virulence determinants associated with outbreak strains [14].

In Tunisia, shigellosis is predominantly caused by *S. flexneri* [15]. However, during 2022–2023, an outbreak of MDR *S. sonnei* was reported in the country, raising significant concerns both nationally and internationally [16].

In this study, we aimed to investigate the genomic characteristics of MDR *S. sonnei* responsible for this outbreak, including their clonal relationship, antimicrobial resistance determinants and relationship to international strains.

## Methods

### Bacterial isolates

Between July 2022 and November 2023, 62 *S. sonnei* isolates were collected at the Laboratory of Microbiology, Habib Bourguiba University Hospital of Sfax, Tunisia. Isolates were mostly obtained from children. Following the 2022 guidelines of the European Committee on Antimicrobial Susceptibility Testing, these isolates were MDR, defined as resistance to at least three different classes of antibiotics. Among them, 24 isolates were randomly selected for WGS, covering each month of the outbreak and each antimicrobial resistance profile.

### WGS, read assembly and quality control

Genomic DNA from the 24 selected *S. sonnei* samples was extracted using the QIAamp DNeasy Blood and Tissue Kit (Qiagen Inc., Valencia, CA). WGS was performed on the Illumina NovaSeq and NextSeq500 platforms using 2×150 bp paired-end chemistry. Subsequently, assembly was performed using Shovill (v.1.1.0) (https://github.com/tseemann/shovill).

### Bioinformatic analysis

#### Genome-based typing and clonal relationship assessment

*In silico* serotyping was conducted using ShigaTyper 2.0.5 to confirm species identification [17]. Seven-locus sequence typing (ST) and core genome multi-locus ST (cgMLST) were conducted using the PubMLST database (https://pubmlst.org/). HC5 and HC10 clusters were inferred based on the *Escherichia/Shigella* v1 scheme available on EnteroBase (https://enterobase.warwick.ac.uk/), utilizing the hierarchical clustering of CgMLST (HierCC) tool [18,19]. HierCC groups genomes into hierarchical clusters (HCs) based on the number of cgMLST allelic differences, with thresholds set at five differences for HC5 and ten differences for HC10. The lineage, clade and subclade of all isolates were determined using Mykrobe (v.0.12.1) based on the framework developed by Hawkey *et al.* [20,21]. Core-genome single-nucleotide polymorphisms (cgSNPs) were identified using Snippy (v.4.6.0) (https://github.com/tseemann/snippy), with *S*. *sonnei* 53G (GenBank accession no. NC_016822) selected as the reference genome. Gubbins (v.3.2.1) [22] was used to identify and mask genomic regions associated with recombination sites. A maximum-likelihood tree was constructed with the IQ-TREE tool (v.2.0.3) [23]. The cgSNP distance between each pair of genomes was determined using PairSNP (https://github.com/gtonkinhill/pairsnp).

#### Resistance determinants analysis

The ResFinder database [24] was employed through the ABRicate tool (v.1.0.1) (https://github.com/tseemann/abricate) to detect the acquired resistance genes to the main antibiotic families, including beta-lactams, macrolides, quinolones, tetracyclines and sulfonamides. Additionally, mutations in genes linked to quinolone resistance (*gyrA* and *parC*) were investigated after executing Mykrobe (v.0.12.1) using Sonneityping (https://github.com/katholt/sonneityping) [20,21].

#### Temporal phylodynamic analysis of outbreak genotype

The temporal signal was confirmed using tip-to-root regression with TempEst (v.1.5.3) and the date randomization test by generating 20 repetitions with the TipDatingBeast R package [25], which was run for 100 million steps. To enhance the temporal signal, the sequences of South Tunisian outbreak strains were supplemented with publicly available sequences from the same HC10 (collection date: before 2022) from EnteroBase [18]. A core-genome alignment of the downloaded assemblies with the South Tunisian sequences was constructed with Snippy. Gubbins was applied to remove recombination sites. To mitigate sampling bias, a maximum of 20 sequences per country per year were selected based on their genetic divergence using the Phylogenetic Diversity Analyzer (v.1.0.3) [26]. The substitution model was evaluated with ModelFinder [27], implemented in IQ-TREE [23], and the K3P model was selected based on the Bayesian information criterion. The Bayesian Skyline model was run in triplicate using a relaxed lognormal clock for 500 million steps using beast (v.1.10.4), ensuring an effective sample size (ESS) >200. Tree and log files were combined and analysed to determine the time to the most recent common ancestor (tMRCA), the maximum clade credibility (MCC) tree and the dynamic of lineages over time. The 95% highest posterior density (HPD) intervals were estimated when applicable.

### International spatiotemporal analysis of the outbreak genotype

All publicly available sequences with the same HC10 as the outbreak strains, available as of March 2024, were retrieved from EnteroBase [18]. Resistance genes were identified using ABRicate with the ResFinder database. The metadata and resistance profile findings of these sequences are detailed in Table S2. In total, 270 sequences were retained and aligned for the Bayesian phylogenetic analysis as described above. The substitution model GTR+F+I model was selected using ModelFinder [27], implemented in IQ-TREE [23].

As detailed in the previous section, after validating the temporal signal was evaluated with TempEst and via date randomization test, the Bayesian Skyline population model was run using beast (v.1.10.4) in triplicate for a total of 500 million steps with a 10% burn-in. Log files with ESS >200 were retained and combined. The MCC tree was visualized using the Interactive Tree Of Life tool [28]. SPREAD3 [10] and StateTransitionCounter (from the Babel v.0.4.2 package, available at https://github.com/rbouckaert/Babel) were used to analyse the spatiotemporal spread and the lineages over time by country, respectively.

## Results

### Clonal relationship

**Fig. 1.**
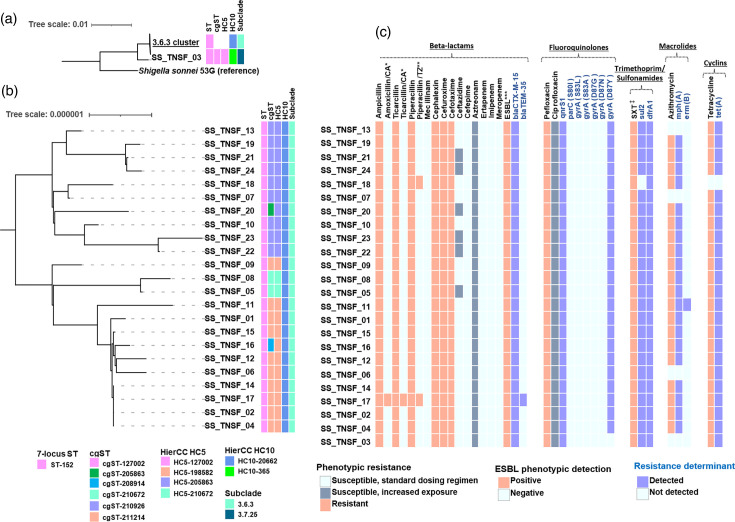
Genomic profiling of the South Tunisian *S. sonnei* strains. (**a**) Maximum-likelihood phylogenetic tree of South Tunisian *S. sonnei* sequences, including the 3.7.25 sequence and the 3.6.3 cluster, alongside the reference sequence. (**b**) Maximum-likelihood phylogenetic tree of the 3.6.3 cluster, encompassing 23 out of the 24 *S. sonnei* sequences (midpoint-rooted tree). Both phylogenetic trees are annotated with heatmaps displaying sequence type (ST), core-genome ST (cgST), hierarchical clustering levels HC5 and HC10 and subclade classifications. (**c**): Heatmap of phenotypic resistance with genomic determinant. The order of sequences was kept the same as in the previous maximum-likelihood tree. *CA: clavulanic acid. **TZ: tazobactam. ***ESBL: extended-spectrum *β*-lactamase. ^‡^SXT: trimethoprim/sulfamethoxazole.

All outbreak *S. sonnei* isolates belonged to sequence type ST-152. Phylogenetic analysis showed that 23 out of 24 strains were closely related genetically, with pairwise cgSNP differences between 0 and 23 (Figs 1a, b and S1). These 23 strains were assigned to five related cgMLST profiles and grouped into a single HC10 cluster (HC10-20662). These isolates belonged to lineage 3, clade 6, subclade 3 (3.6.3, Central Asia III).

The remaining singleton sequence belonged to a different cgMLST and was classified into the HC10-365 cluster. This sequence showed considerable dissimilarity to HC10-20662 sequences. It was classified into lineage 3, clade 7, subclade 25 (3.7.25, MSM4). The pairwise cgSNP distance between the 3.7.25 isolate and the 3.6.3 strains varied between 344 and 358 cgSNPs.

### Antimicrobial resistance determinants

All 24 *S. sonnei* strains harboured resistance determinants to more than three antimicrobial classes (Fig. 1c). All isolates carried the *bla_CTX-M-15_* gene, associated with extended-spectrum *β*-lactamase (ESBL) production, while one isolate additionally contained the *bla_TEM-35_* gene, which was linked with resistance to two beta-lactamase inhibitors: clavulanic acid and tazobactam. Furthermore, all strains harboured the D87Y mutation in the *gyrA* gene and possessed the *qnrS1* resistance gene, which was linked to pefloxacin resistance and reduced susceptibility to ciprofloxacin (median ciprofloxacin minimum inhibitory concentration=0.38 µg ml^−1^). Additionally, the *dfrA1* and sul2 genes responsible for trimethoprim/sulfamethoxazole (SXT) resistance were detected in all sequences except one, which harboured only *dfrA1*. Regarding macrolide resistance, 20 out of 24 isolates carried the *mph(A)* resistance gene, with one sequence additionally harbouring the *erm(B)* gene. Phenotypic antimicrobial testing confirmed the expression of these genes, which was associated with resistance to azithromycin. Genomic analysis also revealed the presence of the *tet(A)* tetracycline resistance gene in most isolates (*n*=23/24).

### Temporal analysis of the Tunisian 3.6.3 (HC10-20662) outbreak strains

**Fig. 2.**
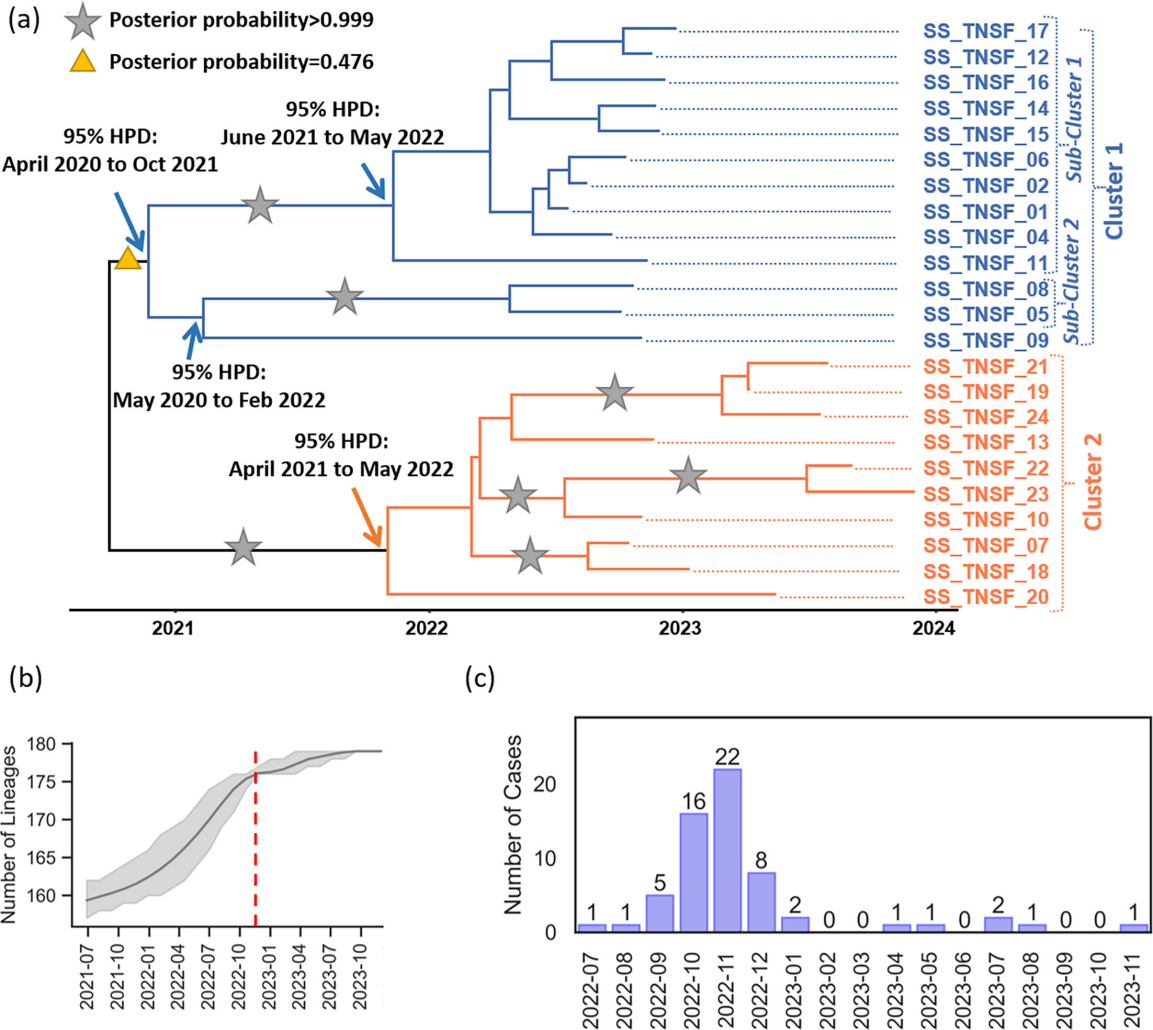
Temporal analysis of the South Tunisian HC10-20662 outbreak strains. (**a**) Time-scaled MCC tree of HC10-20662 strains, with Clusters 1 and 2 highlighted in orange and blue, respectively. The analysis additionally incorporated 147 international sequences collected before 2022 to enhance the temporal signal, though these sequences were excluded from the final visualization. Ninety-five percent HPD refers here to the 95% HPD interval. (**b**) Lineage-through-time plot showing the number of lineages over time. The scale starts from 160 lineages due to the inclusion of international sequences. The red line marks the end of the rapid lineage expansion. (**c**) Monthly number of confirmed *S. sonnei* cases reported by the laboratory of microbiology, Habib Bourguiba University Hospital, Sfax, Tunisia.

Tip-to-root regression analysis and date randomization test (Fig. S2) demonstrated a robust temporal signal, indicating the suitability of genomic data for phylodynamic analysis. The estimated tMRCA for all 3.6.3 (HC10-20662) outbreak isolates was December 2020 (95% HPD interval: December 2019 to August 2021). The Bayesian phylogenetic tree revealed two distinct genetic clusters. Cluster 1 consisted of strains exclusively isolated in 2022 and included two main subclusters, both supported by posterior probabilities exceeding 0.99. These subclusters were estimated to have diverged in January 2021 (95% HPD interval: April 2020 to October 2021). In contrast, Cluster 2 encompassed sequences from 2022 and 2023 and was also strongly supported by a high posterior probability (Fig. 2a). The temporal analysis revealed that the number of *S. sonnei* lineages increased slowly until early 2022 (Fig. 2b). Then, there was a rapid rise in the number of lineages until November 2022, which coincided with the peak of shigellosis cases observed in the region (Fig. 2c). From December 2022 to November 2023, the expansion of these lineages slowed considerably. However, a persistent low-level circulation of the outbreak genotype was noted during this period.

### Intternational spatiotemporal analysis of the HC10-20662 outbreak genotype

A total of 690 publicly available HC10-20662 sequences were analysed. These sequences were all classified into the 3.6.3 subclade. They originated from 16 countries across four continents, with ~90% from the UK (*n*=402) and France (*n*=223). Of note, most sequenced HC10-20662 strains from France (*n*=209) were collected since 2022. The earliest published sequences of the HC10-20622 genotype date back to 2015, originating in the UK and Ireland.

**Fig. 3.**
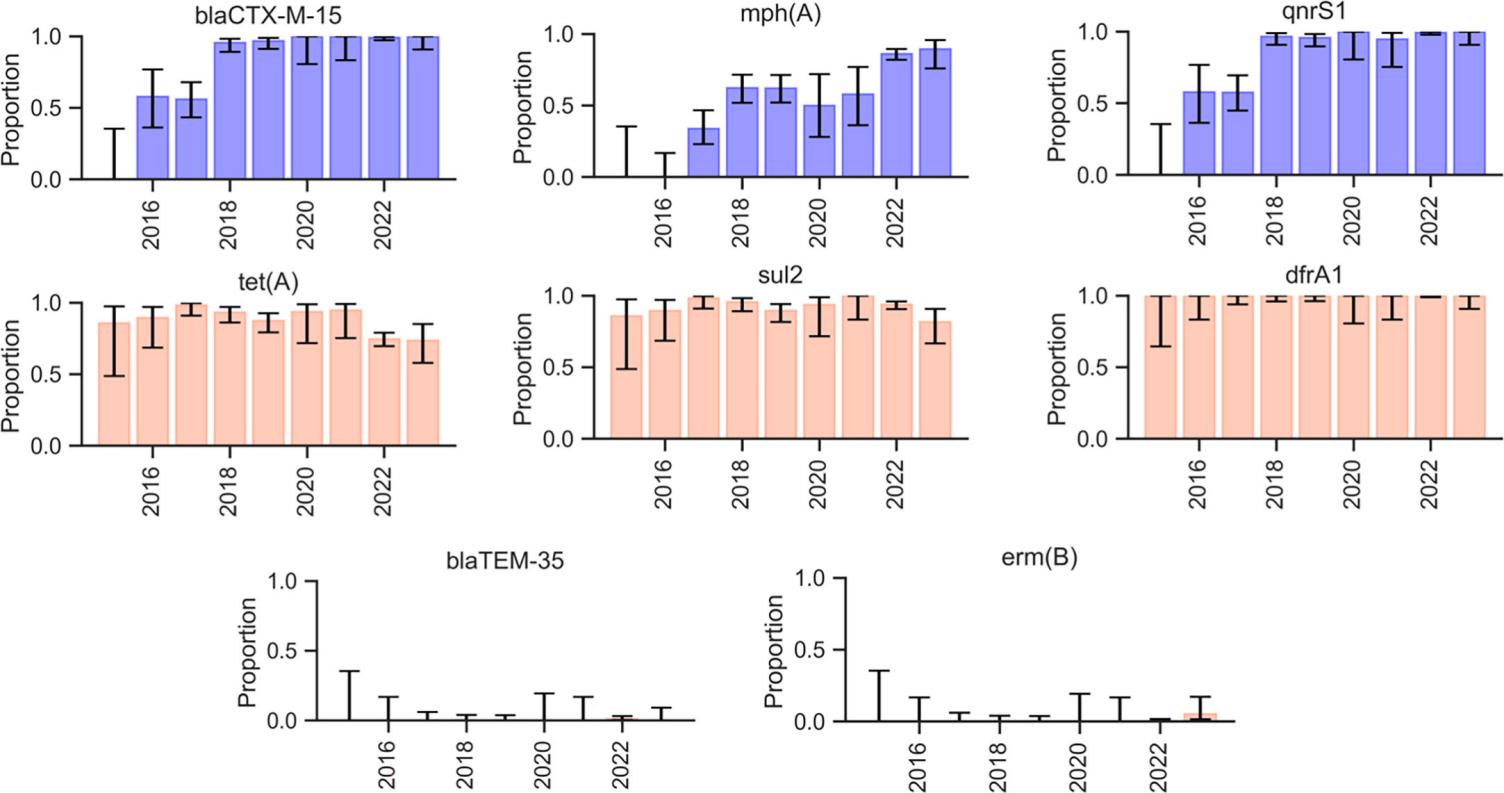
Evolution of resistance genes carriage by the HC10-20662 genotype internationally.

Antimicrobial determinant screening indicated that the proportion of HC10-20662 *S. sonnei* strains harbouring the *bla_CTX-M-15_* and *qnrS1* genes increased rapidly, until reaching almost 100% since 2018. The proportion of strains carrying the *mph(A)* gene increased at a slower manner, until reaching 85–90% during 2022–2023. Conversely, the detection rates of *tet(A)*, *sul2* and *dfrA1* remained always elevated. Of note, the *bla_TEM-35_* and *erm(B)* genes, each observed in a single Tunisian sequence, were mostly undetected in international sequences (Fig. 3).

The tip-to-root regression analysis and date randomization test (Fig. S3) revealed a strong temporal signal. The substitution rate of the HC10-20662 genotype internationally was estimated at 3.8×10⁻^7^ substitution site⁻^1^ year⁻^1^ (95% HPD interval: 3.4×10⁻^7^ to 4.3 x 10⁻^7^ substitution site⁻^1^ year⁻^1^).

**Fig. 4.**
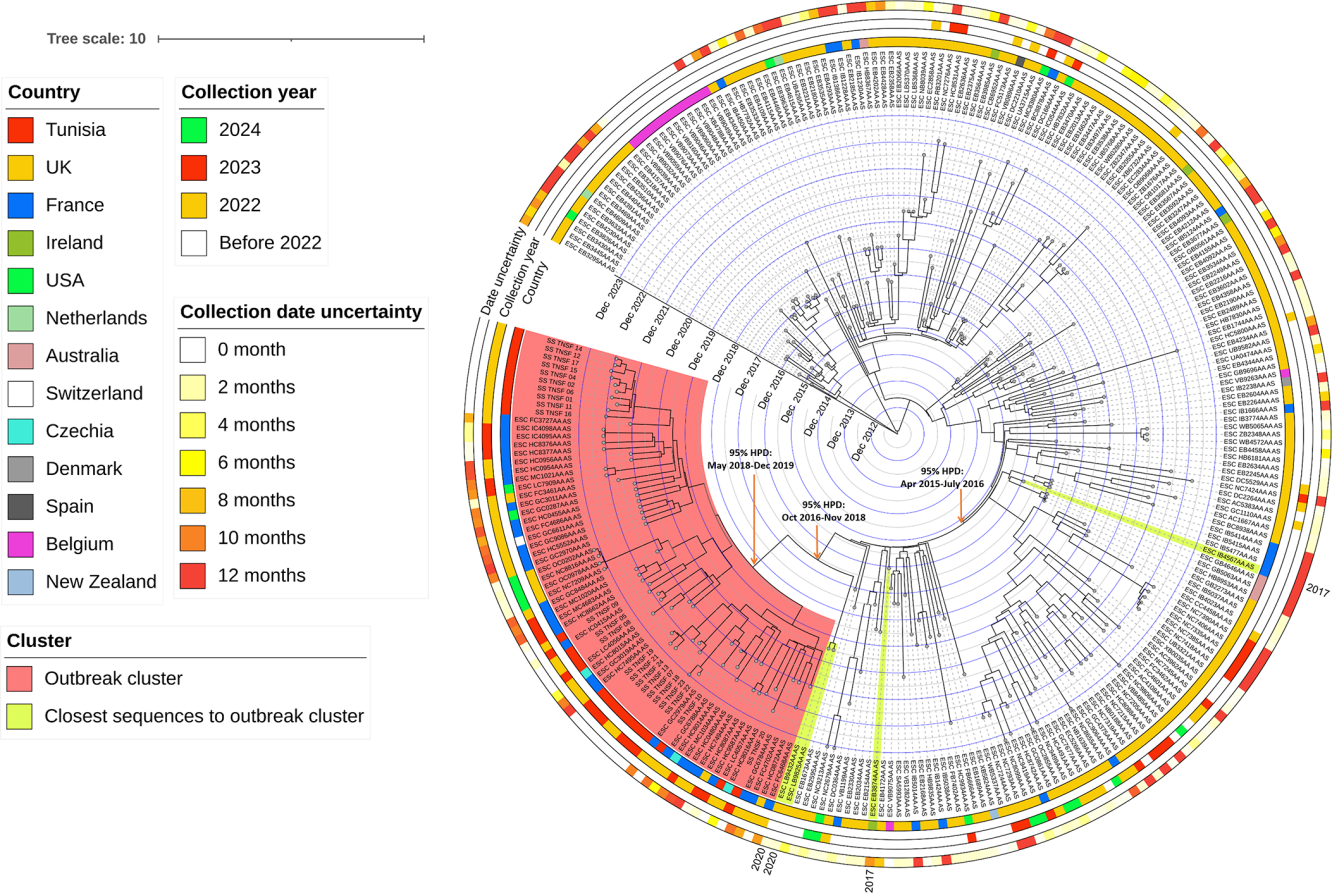
Bayesian time-calibrated phylogenetic tree of the international sequences belonging to the HC10-20662 genotype. The phylogenetic analysis was performed using a representative subset of international sequences (*n*=270). The figure includes a heatmap indicating the country of origin, collection year and uncertainty in collection dates for sequences without precise sampling information (i.e. missing collection day and/or month). For each of these sequences, the actual collection date falls within a time frame that begins with the date indicated by its tip and extends for a duration equal to the associated uncertainty. The sequences most closely related to the outbreak cluster were highlighted in yellow, based on their closest ancestral relationship (inferred through Bayesian phylogenetic analysis) and their genetic proximity (assessed by branch length in maximum likelihood phylogenetic analysis). Ninety-five percent HPD refers here to the 95% HPD interval.

Bayesian (Fig. 4) and maximum-likelihood (Fig. S4) phylogenetic analyses indicated that Tunisian sequences were closely related to sequences from France, forming a distinct outbreak cluster. This cluster consisted solely of strains collected since 2022 and included a few sequences from other countries, such as the UK, Czechia, Switzerland and the USA. The tMRCA of this cluster was estimated to be in March 2019 (95% HPD interval: May 2018 to December 2019).

In terms of shared ancestral history with other HC10-20662 strains, the most closely related strains to the outbreak cluster were collected in the UK in 2020, with a divergence date estimated in November 2017 (95% HPD interval: October 2016 to November 2018). Prior to this, the highest genetic relatedness was observed with sequences of strains collected in 2017 from France and Ireland.

Phylogeographic analysis suggested the widespread circulation of the HC10-20662 genotype in Europe, with subsequent dissemination to other continents, including America, Australia and New Zealand, before being introduced to Tunisia.

**Fig. 5.**
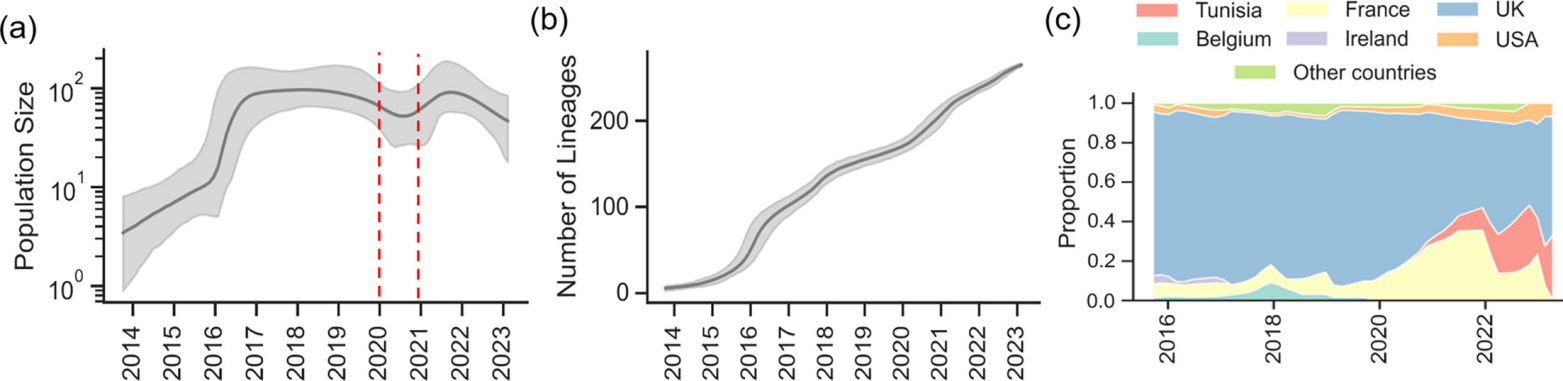
Temporal phylodynamic analysis of the HC10-20662 genotype internationally. (**a**) Bayesian Skyline plot of the HC10-20662 genotype internationally. (**b**) Lineage-through-time plot of the HC10-20662 genotype globally. (**c**) Lineage-through-time plot of the HC10-20662 genotype by country.

Temporal analysis revealed that the global HC10-20662 *S. sonnei* population increased until reaching a peak between 2017 and 2019, followed by a decline in 2020. Then, a resurge has been observed in several countries. Specifically, the number of lineages increased consistently until reaching a peak in late 2022 in both France and Tunisia (Fig. 5).

## Discussion

Shigellosis poses a significant global public health concern, with *S. flexneri* and *S. sonnei* being responsible for the majority of cases worldwide [29]. In Tunisia, akin to other low- and middle-income countries, *S. flexneri* has historically been the prevailing species, while cases of *S. sonnei* were less frequent [15,30]. Shigellosis has generally remained sporadic in the country, with the Reference Laboratory at the Pasteur Institute of Tunis reporting between one and seven confirmed cases annually during 2012–2021 [31]. However, a notable uptick in Shigella cases caused by MDR *S. sonnei* was noted in many regions across the country during 2022. The first confirmed cases of MDR *S. sonnei* were reported back in June 2022. The number of cases has continued to escalate until reaching a peak in November 2022. As of 8 January 2023, the National Observatory of New and Emerging Diseases has reported 339 confirmed cases in Tunisia, including one hospital fatality. The observatory also reported that environmental investigations were conducted to search for *S. sonnei* in water, food and surfaces (*n*=232 samples as of 1 February 2023), but all results were negative, and the source of the outbreak remained unidentified [31].

This outbreak highlights a notable shift in the epidemiology of shigellosis. While prevalent in developed nations, *S. sonnei* is becoming more frequently observed in developing regions across Asia, Latin America, the Middle East [32,33] and, as reported by this study, North Africa. This emphasizes the dynamic nature of shigellosis transmission and underscores the critical importance of continuous surveillance and adaptation of public health strategies.

Our findings revealed that all studied Tunisian isolates belonged to the same lineage 3. It has been previously reported that lineage 3 is the most frequent *S. sonnei* lineage worldwide. Within this lineage, Hawkey *et al.* showed that the clades 3.6 and 3.7 were the most common overall, representing 19 and 62% of all available sequences as of 2018, respectively [20].

In our cohort, 23 out of 24 sequenced isolates belonged to the 3.6.3 subclade. These isolates exhibited close genetic similarity, with few differences in pairwise cgSNPs and cgMLST loci, and were grouped within the HC10-20662 genotype. These isolates were obtained from various regions in South Tunisia.

Phylodynamic analysis revealed that this genotype continued to grow rapidly until the end of 2022, resulting in the shigellosis outbreak observed in the country during this period. Subsequently, the growth rate of the HC10-20662 outbreak genotype largely declined in 2023. However, it continued circulating at a low level, which is likely facilitated via undetected cases and asymptomatic carriers.

Comparative analysis with international sequences revealed that the HC10-20662 genotype within the 3.6.3 subclade was predominantly detected in Europe. It is noteworthy that while the 3.6.3 subclade is part of the Central Asian expansion of lineage III, historically associated with regions in Central Asia, our investigation did not identify any Asian sequences corresponding to HC10-20662 within the EnteroBase database, except for 14 Russian sequences, which were excluded from the phylogeographic analysis due to the unavailability of collection year. Moreover, there was no evidence of travel to Central Asia among Tunisian cases included in this study.

Phylodynamic analysis indicated that the substitution rate of international HC10-20662 strains was close to the rates reported from previous studies examining *S. sonnei* [34,36]. Phylogeographic analysis suggested that the HC10-20662 genotype initially emerged and spread within high-income European countries until 2019. Then, a decrease in the circulation of the HC10-20662 genotype was observed during 2020 and early 2021, which coincided with the onset of the COVID-19 pandemic, marked by widespread lockdowns and public health measures. This decline mirrors the broader trend observed for *S. sonnei* by the European Centre for Disease Prevention and Control (ECDC), which reported a reduction in *S. sonnei* circulation during the same period [37].

Considering this epidemiological context and the tMRCA estimation for Tunisian sequences (December 2019 to August 2021), it is likely that the introduction of the outbreak genotype to Tunisia occurred either prior to the onset of the pandemic or following the relaxation of public health measures in 2021. The most probable source of importation is a European country, as strains with the closest ancestral phylogenetic relationship to the outbreak cluster were collected from the UK in 2020. However, tracing the source of importation is complicated by the limited number of *S. sonnei* sequences from multiple underrepresented countries and the uncertainty surrounding the collection dates of many publicly available sequences, which lack precise information regarding the day and, in some cases, the month of collection. Additionally, it is difficult to ascertain the location without travel information for the international genomes. This issue is particularly relevant since a considerable proportion of shigellosis cases reported in European countries are linked to travel to other regions [10,37], which makes it hard to determine whether a specific genome represents a local case or an imported case.

By late 2022, phylogeographic analysis unveiled a significant upsurge in the prevalence of the HC10-20662 clade across various countries. This was associated with the expansion of the outbreak cluster containing sequences from Tunisia and different European nations including France, the UK, Czechia and Switzerland. This observation aligns with the ECDC report, which indicated that a multinational outbreak was associated with this genotype, with many cases being linked with travel to Tunisia [37]. International travel has potentially facilitated the spread of HC10-20662 strains between countries, particularly after the lifting of COVID-19-related restrictions.

Conversely, a single Tunisian isolate stood out genetically and was categorized within the 3.7.25 genotype, identified as MSM4. Typically, this subclade is linked to men having sex with men in European nations. However, there are insufficient epidemiological data available regarding this case.

In terms of antimicrobial resistance, the 2022–2023 outbreak in Tunisia marked the first instance of an MDR *S. sonnei* outbreak in the country, to the best of our knowledge. MDR in *Shigella* spp. is a global public health problem. The emergence of MDR * S. sonnei* outbreaks, as reported in this paper, aligns with the global trend of increasing antimicrobial resistance within this species [38,39]. Most isolates in this study were characterized by resistance to multiple antimicrobial families, including beta-lactams, macrolides, tetracycline and SXT, as well as reduced susceptibility to ciprofloxacin.

The similarity of phenotypic and genotypic resistance profiles among Tunisian isolates belonging to different genotypes suggests a potential horizontal transfer of resistance plasmids between the isolates of the 3.6.3 subclade and the 3.7.25 strain.

In particular, beta-lactams resistance via ESBL production was associated with *bla_CTX-M-15_* in the studied strains, which was also observed in international HC10-20662 strains. The increasing trend of ESBL-producing *S. sonnei* was described in other subclades. For instance, a recent study from France revealed that *bla_CTX-M-15_* was frequently detected in many *S. sonnei* subclades, including 3.6.1, 3.6.1.1 and 3.6.1.1.2 [40].

Of note, one Tunisian strain displayed resistance to beta-lactamase inhibitors through the carriage of *bla_TEM-35_*, which was identified in a child treated with amoxicillin/clavulanic acid in the previous 5 days. The co-carriage of both *bla_CTX-M-15_* and *bla_TEM-35_* genes was previously documented in *S. sonnei* [41]. However, this association was not detected in the majority of H10-20662 strains internationally.

In addition, most isolates carried the *mph(A)* gene, which confers resistance to azithromycin, a recommended antibiotic for the treatment of shigellosis [42]. All Tunisian isolates displayed reduced susceptibility to fluoroquinolones. In general, fluoroquinolone resistance typically arises from the accumulation of mutations occurring in the quinolone resistance-determining region of DNA gyrase (*gyrA* and *gyrB*) and topoisomerase IV (*parC* and *parE*) genes, as well as plasmid-mediated quinolone resistance genes, such as *qnrA, qnrB, qnrC, qnrD, qnrS, qepA* and *aac(6′)-Ib-cr* [43,44]. In the sequenced isolates, only the *qnrS1* gene and the D87Y mutation in *gyrA* were identified, which is concordant with phenotypic reduced susceptibility to ciprofloxacin. The median ciprofloxacin MIC of the Tunisian strains in particular, as well as the overall phenotypic and genotypic resistance profiles in general, were highly similar to data reported in France during 2022 according to the ECDC report, further highlighting the relatedness of strains from both countries [45].

## Conclusion

Overall, we present the genomic profile of outbreak *S. sonnei* strains isolated in South Tunisia between 2022 and 2023. To the best of our knowledge, this is the first report of an outbreak caused by MDR *S. sonnei* in the country. Our investigation revealed the predominance of the HC10-20662 genotype within the 3.6.3 subclade, as the main genotype driving the outbreak. Ongoing genomic surveillance initiatives at both the national and international levels are crucial to monitor the dynamic evolution and global spread of the HC10-20662 outbreak genotype. These surveillance efforts should provide valuable insights that enable public health authorities to implement targeted interventions to minimize the potential impact of the outbreak. Furthermore, the MDR nature of the outbreak isolates underscores the urgent need to combat antimicrobial resistance, including prudent antimicrobial use and raising awareness to reduce self-medication practices.

## supplementary material

10.1099/mgen.0.001362 Uncited Supplementary Material 1.

10.1099/mgen.0.001362 Uncited Supplementary Material 2.

## Abbreviations

cgMLST: core genome multi-locus sequence typing
cgSNPs: core-genome single nucleotide polymorphisms
ECDC: European Centre for Disease Prevention and Control
ESBL: extended spectrum *β*-lactamase
ESS: effective sample size
HCs: hierarchical clusters
HierCC: hierarchical clustering of CgMLST
HPD: high posterior density
MCC: maximum clade credibility
MDR: multidrug-resistant
ST: sequence typing
SXT: trimethoprim/sulfamethoxazole
tMRCA: time to the most recent common ancestor
WGS: whole-genome sequencing
