# Supplementary material for: Molecular epidemiology of a multidrug-resistant Shigella sonnei outbreak in Tunisia (2022–2023) using whole-genome sequencing
Source: Microb Genom. 2025 Mar 6;11(3):001362. doi: 10.1099/mgen.0.001362 (PMC11936343; doi:10.1099/mgen.0.001362)
Supplement: Uncited Supplementary Material 1. [file mgen-11-01362-s001.pdf]

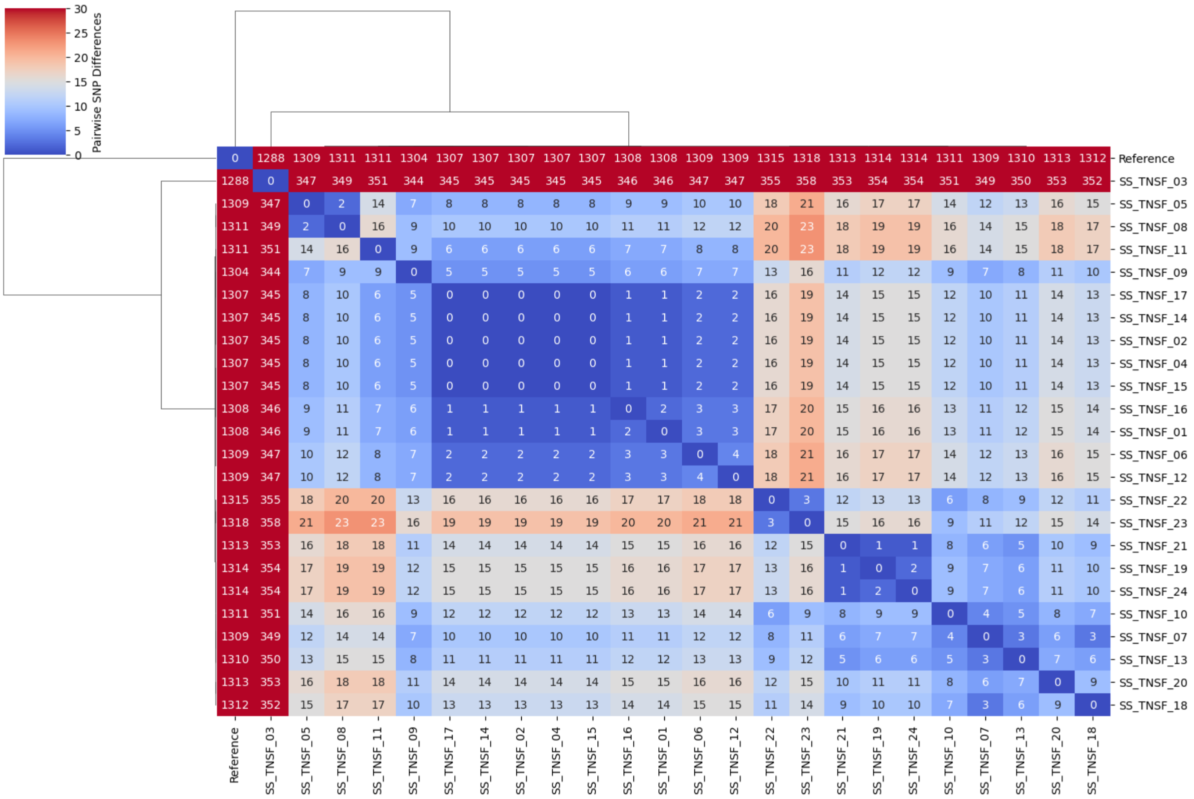

Fig. S1. Pairwise core-genome SNP differences between South Tunisian *S.sonnei* sequences after removing recombination sites.

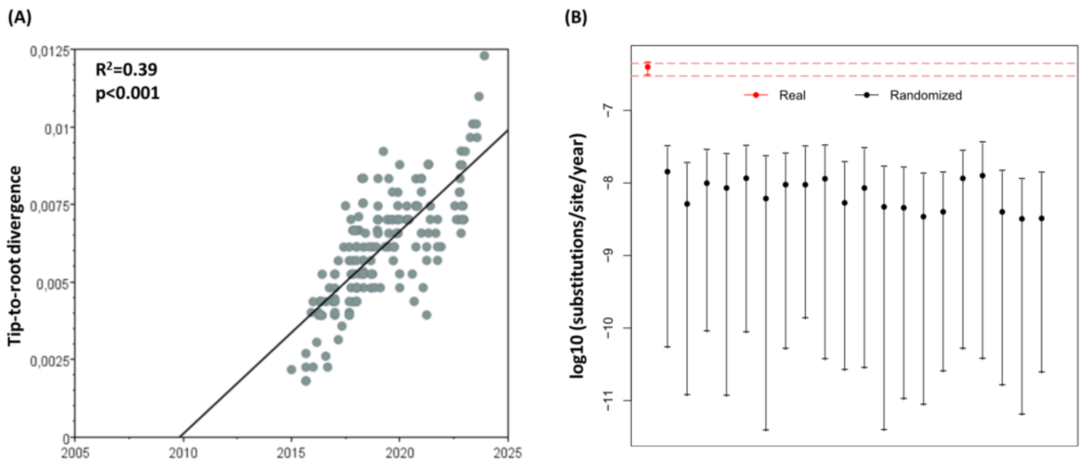

Fig. S2. Temporal signal assessment preceding phylodynamic analysis of South Tunisian *Shigella sonnei* belonging to the HC10-20663 genotype.

To strengthen the temporal signal, 147 additional international sequences from strains isolated before 2022 were incorporated.

A: Tip-to-root regression analysis generated using TempEst. Pearson correlation p-value and R^2 *displayed in the plot.*

B: Date-randomization test conducted with the PlotDRT function in the TipDatingBeast R package.

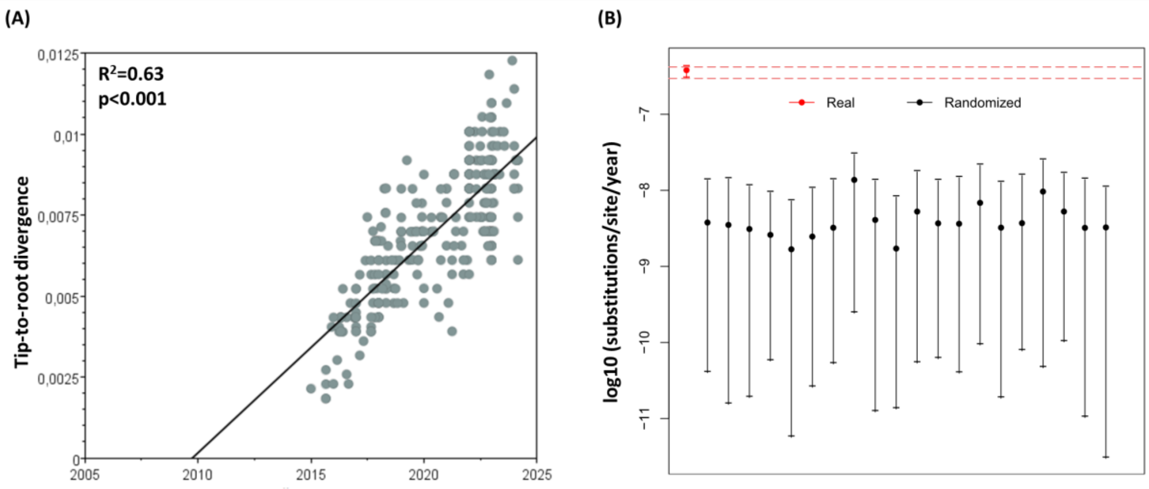

Fig. S3. Temporal signal assessment preceding phylodynamic and phylogeographic analyses of the HC10-20663 genotype internationally.

A: Tip-to-root regression analysis generated using TempEst. Pearson correlation p-value and R^2 displayed in the plot.

B: Date-randomization test conducted with the PlotDRT function in the TipDatingBeast R package.

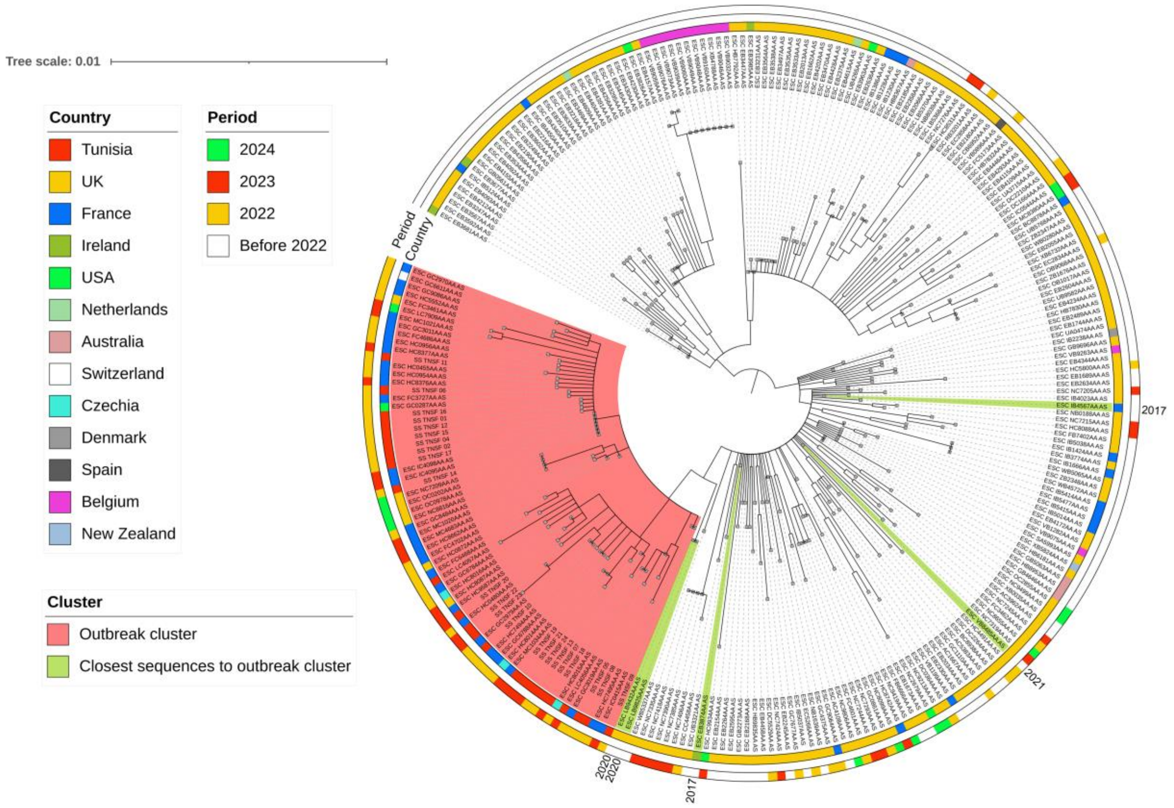

Fig. S4. Maximum likelihood phylogenetic tree of international HC10-20662 sequences

The outbreak cluster is highlighted in red. The sequences with the highest genetic relatedness to the outbreak cluster are highlighted in green.
